# Recent Advances in Vaccine Technology and Design

**DOI:** 10.3390/vaccines10040624

**Published:** 2022-04-15

**Authors:** Rossella Cianci, Laura Franza

**Affiliations:** 1 Department of Translational Medicine and Surgery, Catholic University of Sacred Heart, Fondazione Policlinico Universitario A. Gemelli IRCCS, 00168 Roma, Italy; 2 Emergency Medicine Unit, Catholic University of Sacred Heart, Fondazione Policlinico Universitario A. Gemelli IRCCS, 00168 Roma, Italy; cliodnaghfranza@gmail.com

If up until three years ago, infectious diseases were a lesser concern when compared to non-communicable diseases in Western countries, the ongoing pandemic has reminded us that things are not so clean-cut. The pandemic has indeed highlighted our vulnerability to emerging infective diseases [1], underlining the importance of identifying and promptly treating potential threats.

The most efficient measure against infectious disease remains vaccination, and progress is being made constantly in designing vaccines that are both safe and efficient. Recently, the great advances made in bioinformatic sciences have led to in silico approaches for the development of novel and, potentially effective, vaccine candidates against different pathogens, such as viruses, bacteria and parasites [2]. 

Thus, immunoinformatic methodologies, due to their reliability, safety, low cost, stability, precision and, above all, speed, have recently been used to progress multiple epitope vaccines (MEVs) [3]. MEVs represent an interesting novel approach in designing vaccines: it is sometimes difficult to identify a single antigen capable of determining an immune response and this has been a major problem in different occasions. 

Moreover, immunoinformatics can help detect T-cell/B-cell epitopes, highlight antigenic immunodominant epitopes, reduce allergenicity and toxicity and enhance stability of a vaccine candidate [4]. Yet, it is also worth noting that, after having designed MEVs, a subsequent strong validation in animal models is needed to confirm the real immunogenicity and safety of the vaccine.

In this Special Issue, we have collected papers in which researchers, through the use of immunoinformatics, have designed MEV candidates to fight new and old ubiquitous serious infectious diseases.

Suleman et al. [5], for instance, have used bioinformatics to design a new multi-epitopes subunit vaccine candidate that could potentially determine a strong immune response against an emerging Tick-Borne Encephalitis Virus (TBEV), a member of the Flaviviridae family that has caught the attention of researchers, as it is the causative agent of a severe neurological disease, which is endemic in Eurasia, and transmitted by ticks. 

Rehman et al. [6] have designed a MEV as a new option for Schistosomiasis, which is the second most common tropical disease after malaria. 

The combination of reverse vaccinology, immuno-informatics and subtractive genomics was utilized by Aldakheel et al. in the case of infections by *Clostridium perfringens,* which is a Gram-positive anaerobic bacterium involved in several human and animal diseases. The designed MEV has been proven to offer an interesting and effective immune stimulation. The result is exciting, given that, at the moment, the only available treatment for *Clostridium perfringens* is supportive care, thus if the vaccine is proved safe and efficient in vivo, it will have a great impact on clinical practice [7].

MEV could also help fight multi-resistant nosocomial bacteria, another growing concern in terms of infectious diseases. The genus Klebsiella is among the pathogens showing extended antibiotic resistance [8], and vaccines could be a possible way to treat it, since, up until now, a licensed vaccine is not still available [9]. In the study by Allemailem [10] a computational vaccine design approach was used on the genome of the Klebsiella species. The TonB-dependent siderophore receptor and siderophore enterobactin receptor FepA proteins have been mapped to develop a multi-epitope peptide vaccine construction that has shown a good immunogenicity. Moreover, a potential effective multiple epitope subunit vaccine against *Klebsiella aerogenes,* a Gram-negative bacterium, which is difficult to treat, has been identified through a subtractive proteomics and immuno-informatics approach by Umar et al. [11]. 

*Alcaligenes faecalis* is another multi-resistant bacterium creating some concern, given its resistance to almost all antibiotics available to date. The use of a whole proteome-based therapeutic targets annotation has allowed Alharbi and colleagues to design two different multi-epitope vaccine constructs that have shown an immune response through molecular docking [12]. 

An immunoinformatics approach to designing a MEV has also been used in the case of *Providencia rettgeri*, another multi-resistant pathogen, which commonly causes traveller’s diarrhoea [13]. The vaccine designed by Gul et al. showed robust interaction with the immune receptors.

A reverse vaccinology approach has also been used to perform a multi-epitope vaccine against a viral zoonosis, more specifically the rodent-borne hantaviruses, which are a significant social and economic burden on communities [14]. Moreover, docking studies have revealed that this construct could elicit cellular and humoral immune responses.

In the last years, vaccine scepticism has grown, particularly using the idea that vaccination is not a safe medical procedure [15]. While it is obvious that these claims are mostly false, there have been cases of vaccines causing significant morbidity. The contamination of oral poliovirus vaccine with simian virus 40 (SV40) is one of the most known incidents [16]. Live vaccines are indeed particularly vulnerable to contamination, and it is not always easy to identify this, yet Manukyan et al. have been focusing on this particular issue and have found new ways to further improve the safety of vaccines designing a very sensitive assay for Sabin 2 contamination in novel Oral Polio Virus type 2 (nOPV2) vaccine [17]. 

Vaccination campaigns usually target whole groups, such as children or people living in at risk areas, or populations as a whole as seen during the pandemic. Yet not everyone responds in the same way: some people do not respond appropriately to vaccinations, as seen in the cases of hepatitis B, and the mechanisms are not completely clear [18]. The global vaccination effort that took place during the Coronavirus pandemic has offered an ideal scenario to explain the differences between responders and non-responders. For this reason, Colucci et al. observed that genetic background and immune profiles are key factors in determining the differences in the immunoresponse to the BNT162b2 (Pfizer–BioNTech) vaccine, identifying different single-nucleotide polymorphisms (SNPs) of a specific region internal to the 3′Regulatory Region-1 (3′RR1) of the human immunoglobulin constant-gene (IgH) locus [19].

The immune system plays a crucial role in the fight against infectious diseases. The genetic predisposition together with the immune reactions orchestrate an effective response to pathogens; vaccines are able to elicit the production of an adequate acquired immunity. The recent advances in immunoinformatics have paved the way for rapid identification of novel vaccine candidates that could evoke a strong immune response. Moreover, the technological advances that are taking place may allow us to target a number of infectious diseases that, at present, can be treated only through supportive care. This is particularly interesting when considering multi-resistant pathogens, which represent a serious threat globally. Furthermore, the progress that has been made may also allow us to make existing vaccines more efficient and even safer, which is particularly important given the present distrust towards vaccinations.
